# Renal progenitors derived from human iPSCs engraft and restore function in a mouse model of acute kidney injury

**DOI:** 10.1038/srep08826

**Published:** 2015-03-06

**Authors:** Barbara Imberti, Susanna Tomasoni, Osele Ciampi, Anna Pezzotta, Manuela Derosas, Christodoulos Xinaris, Paola Rizzo, Evangelia Papadimou, Rubina Novelli, Ariela Benigni, Giuseppe Remuzzi, Marina Morigi

**Affiliations:** 1 IRCCS - Istituto di Ricerche Farmacologiche Mario Negri, Centro Anna Maria Astori, Science and Technology Park Kilometro Rosso, 24126 Bergamo, ITALY; 2 Unit of Nephrology and Dialysis, Azienda Ospedaliera Papa Giovanni XXIII, 24127 Bergamo, ITALY; 3 Fondazione IRCCS - Policlinico San Matteo, 27100 Pavia, ITALY

## Abstract

Acute kidney injury (AKI) is one of the most relevant health issues, leading to millions of deaths. The magnitude of the phenomenon remarks the urgent need for innovative and effective therapeutic approaches. Cell-based therapy with renal progenitor cells (RPCs) has been proposed as a possible strategy. Studies have shown the feasibility of directing embryonic stem cells or induced Pluripotent Stem Cells (iPSCs) towards nephrogenic intermediate mesoderm and metanephric mesenchyme (MM). However, the functional activity of iPSC-derived RPCs has not been tested in animal models of kidney disease. Here, through an efficient inductive protocol, we directed human iPSCs towards RPCs that robustly engrafted into damaged tubuli and restored renal function and structure in cisplatin-mice with AKI. These results demonstrate that iPSCs are a valuable source of engraftable cells with regenerative activity for kidney disease and create the basis for future applications in stem cell-based therapy.

Acute kidney injury (AKI) is an important cause of morbidity and mortality worldwide affecting more than 13 million people, especially in poor resource countries^1^,^2^. Here, AKI is generally a disease of young adults and children who continue to die as a consequence of this disorder because renal replacement therapy is not available. Therefore the development of new strategies directed to support and improve renal function is greatly demanded. One promising approach aimed at kidney regeneration is cell-based therapy that requires the availability of cells able to efficiently replace cellular components of the diverse renal compartments. Studies have suggested renal progenitors, isolated from adult tissue^3^,^4^,^5^,^6^, as a possible option, although their clinical translation is limited by the inaccessibility of the cell-source in patients, poor expansion capability and restricted differentiation potential. iPSCs, pluripotent cells that possess extensive self-renewal capacity, offer the possibility of patient-specific treatments and may represent a cell type worth investigation for therapy. Undifferentiated murine iPSCs have been described to attenuate kidney injury in ischemia reperfusion-induced AKI only when administered via intra-arterial route, by paracrine action^7^. However, the pluripotent nature of iPSCs raise concerns on high risk of tumour development when these cells are administered without pre-differentiation. An attractive alternative consists in the derivation of renal progenitors from iPSCs. Initial approaches with murine embryonic stem cells (ESCs) and iPSCs have shown the feasibility to generate cells expressing intermediate mesoderm (IM) and MM markers^8^,^9^,^10^,^11^,^12^,^13^,^14^,^15^,^16^,^17^,^18^,^19^,^20^,^21^,^22^ by exposure to specific factors including Activin A and retinoic acid (RA) in combination with bone morphogenic proteins (BMPs) or GDNF, important factors in kidney development^23^. Recently, human ESCs and iPSCs have been described to differentiate into nephrogenic cells, renal proximal tubular and podocyte lineages^16^,^17^,^18^,^19^,^20^,^21^,^22^,^24^. However, robust engraftment of human iPSC-derived RPCs in an animal model of AKI associated with restoration of kidney function and structure has never been shown.

## Results

**Figure 1:**
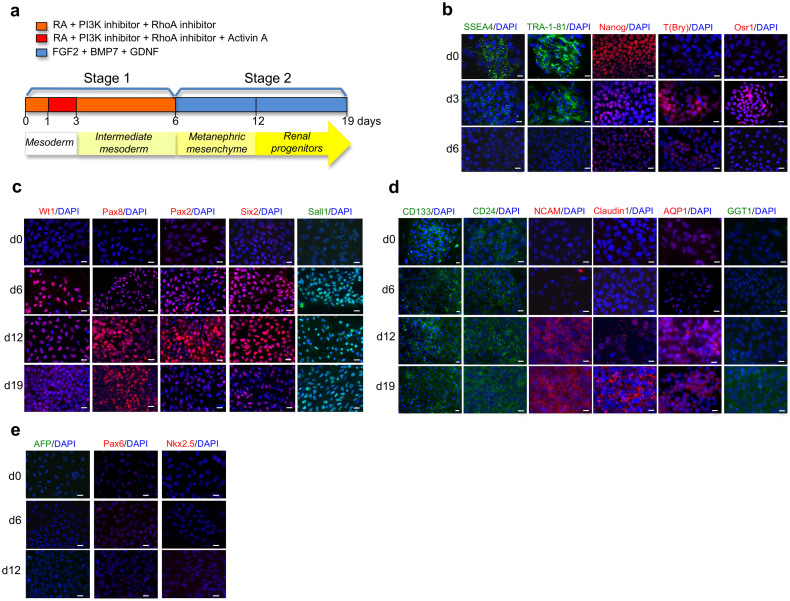
Stepwise differentiation of human iPSCs towards renal progenitor cells (RPCs). (a) Schematic description of the two-stage protocol applied to iPSC-renal commitment. (b, c, d, e) Immunofluorescence of iPSCs (derived from retroviral transfected dermal fibroblasts) exposed to differentiating media. (b) The pluripotency markers SSEA4, TRA-1-81, Nanog, ME marker T(Bry) and IM marker Osr1. (c) IM and MM marker expression as Wt1, Pax8, Pax2, Six2 and Sall1. (d) Renal progenitor markers CD133, CD24, NCAM, glomerular epithelial marker Claudin1 and proximal tubular epithelial markers AQP1, GGT1. (e) Markers identifying endodermal AFP, ectodermal Pax6, and cardiac mesodermal Nkx2.5 derivation. Nuclei are stained with DAPI (blue). Scale bars: 20 μm (b, c, d, e).

Here, we report an efficient and reproducible two-stage protocol to obtain renal progenitors from human iPSCs (Fig. 1a). Initially, we performed differentiation studies on commercially available iPSCs obtained from human foreskin fibroblasts transfected with retrovirus encoding for *Oct4*, *Sox2*, *Klf4*, and *c-Myc*. In stage 1, to induce differentiation toward mesoderm (ME) and IM-like cells, we exposed undifferentiated iPSCs for 6 days to RA and to the small molecules RhoA inhibitor (CCG1423) and PI3K inhibitor (LY294002). The employment of small molecules has been suggested as a simple and direct method to address stem cell differentiation^22^,^25^. Activin A, known to stimulate the expression of IM markers in ESCs^8^,^14^, was added for two days starting on day 2. In stage 2, by day 6 up to 19, iPSCs were exposed to a cocktail of three nephrogenic factors FGF2, BMP7 and GDNF to commit cells towards MM. Undifferentiated iPSCs expressed pluripotency markers SSEA4, TRA-1-81, and Nanog (Fig. 1b) that progressively decreased after exposure to stage 1-medium. SSEA4 and TRA-1-81 were almost completely absent on day 6 whereas only few cells expressed a weak Nanog staining (Fig. 1b). By cytofluorimetric analysis, when we analyzed undifferentiated iPSCs, we found that the mean percentage of cells expressing TRA-1-81 was 88.5% ± 0.9 whereas it decreased at 24.7% ± 1.39 and 22.2% ± 0.93 respectively on day 12 and 19 of differentiation, exhibiting a very low fluorescence intensity per cell. As early as 3 days, we detected the expression of T (Brachyury), a transient marker of ME, that decreased on day 6 to a faint signal (Fig. 1b). Osr1, one of the earliest transcription factors specifying a multipotent population of IM cells giving rise to MM cells^26^, increased after 3 days and progressively decreased at 6 days (Fig. 1b). Other IM-specific markers including Wt1, Pax8 and Pax2 were observed by day 6 (Fig. 1c). At the same time, we found initial expression of Six2 and Sall1 (Fig. 1c) indicating the establishment of precursor MM fate. As a result of the exposure to stage 2-medium, on day 12 we found stable expression of Wt1, Pax8, Pax2, Six2 and Sall1 that decreased on day 19 except for Sall1 that maintained high expression. The developmental progression towards renal progenitor phenotype was confirmed by the presence of specific markers such as NCAM, CD133 and CD24 that during differentiation acquired cellular-specific localization. NCAM, normally expressed in the nephrogenic zone of developing kidney^27^ was induced by day 12 showing a clear pattern of expression at the cell periphery (Fig. 1d). CD133 and CD24, markers found in MM cells and glomerular progenitor cells^28^,^29^, were expressed on almost all the cells at any differentiation time considered (Fig. 1d) however, their distribution changed from a punctuated staining in undifferentiated iPSCs to a characteristic well-organized peripheral pattern by day 12 (Fig. 1d). Finally, we characterized the expression of Claudin1, a marker of glomerular epithelial cells and Aquaporin 1 (AQP1) and gamma-glutamyl transferase 1 (GGT1) markers of proximal tubular cells. By day 12, we observed a weak induction of Claudin1 and an intense AQP1 staining (Fig. 1d) whereas GGT1 was weakly expressed on day 19 (Fig. 1d). Differentiated iPSCs did not express alpha-fetoprotein (AFP), Pax6 and Nkx2.5, markers of endoderm, neuroectoderm and cardiac mesoderm, respectively (Fig. 1e).

**Figure 2:**
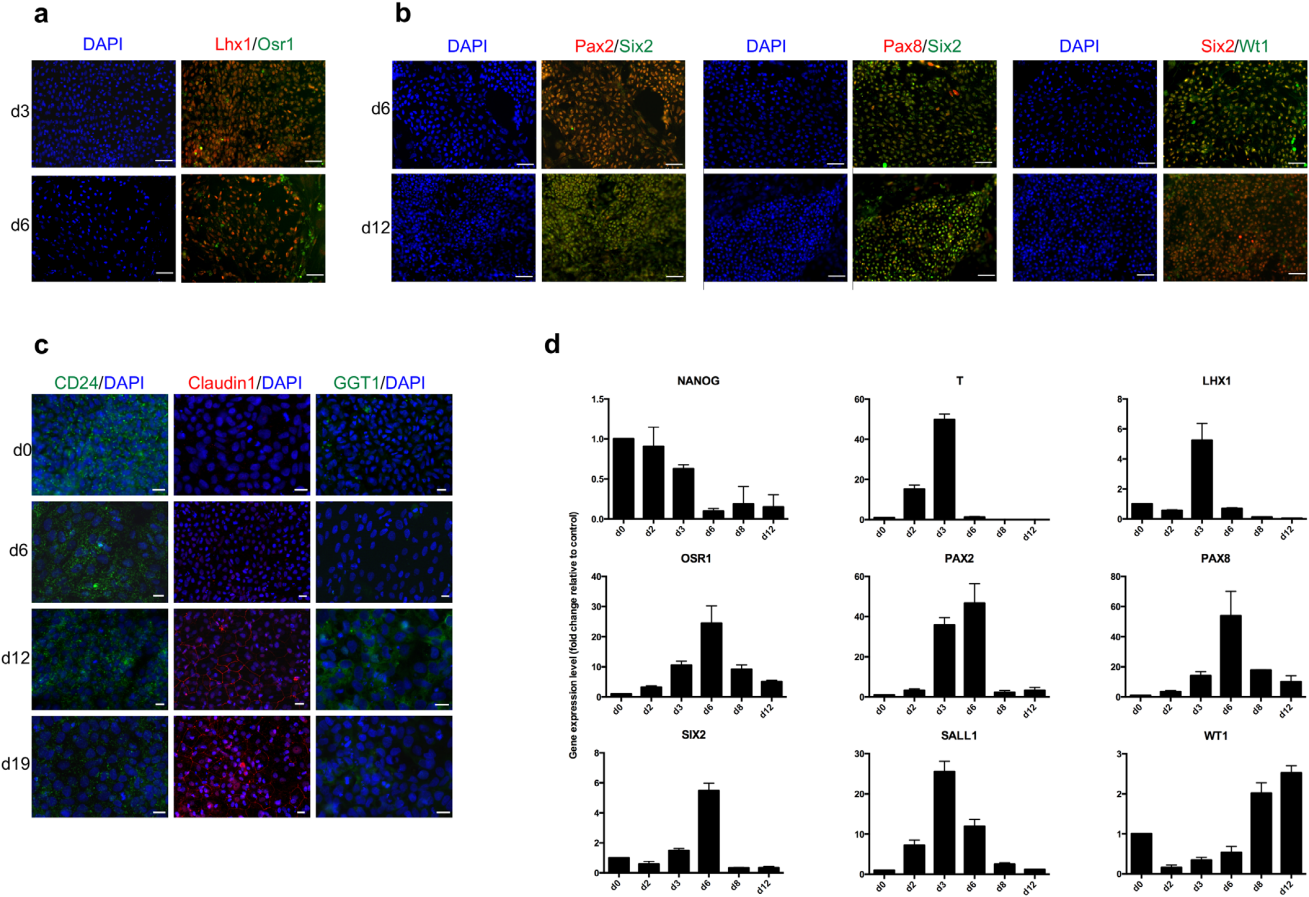
Differentiation of human iPSC clone IV towards renal commitment. (a) Representative immunofluorescence images of co-staining for Lhx1/Osr1 up to day 6. (b) Images of co-staining of Pax2/Six2, Pax8/Six2 and Six2/Wt1 from day 6 to 12. (c) Expression of renal progenitor markers such as CD24, Claudin1 and GGT1 from day 0 to 19. Nuclei are stained with DAPI (blue). Scale bars: 50 μm (a, b), 20 μm (c). (d) Gene expression analysis for renal progenitor markers at different points in time.

Next, we tested the robustness of the two-step differentiation protocol on iPSCs generated in our laboratory by reprogramming human fibroblasts using the STEMCCA Cre-excisable polycistronic lentivirus with excisable reprogramming cassette. Specifically, the generated iPSC clones showed all the properties of the pluripotent stem cells in terms of expression of pluripotency markers, karyotype and ability to differentiate both *in vitro* and *in vivo* into derivatives of the three germ layers (Supplementary Fig. 1a-f). The fully characterized clone IV was used to test the differentiation protocol. Immunofluorescence analysis showed a pattern of protein expression similar to that observed with commercial iPSCs. Indeed, downregulation of the pluripotency marker Nanog was observed going from undifferentiated cells to day 6 of differentiation. Conversely, upregulation of markers specific to the early ME, which included T, for the IM such as Lhx1, Osr1 (Supplementary Fig. 2a) and later Wt1, Pax8 and Pax2, and for the MM such as Six2 and Sall1 was detected (Supplementary Fig. 2b). To better define the phenotype of iPS clone IV-derived RPCs, we evaluated the co-expression of several markers which defines renal commitment. Our data show the specification into a homogeneous IM cell population from day 3 to 6 as demonstrated by Lhx1/Osr1 co-expression (Fig. 2a). Six2 co-staining with Pax2, Pax8 and Wt1 at day 6 and 12 confirms differentiation toward MM cell phenotype (Fig. 2b). Protein expression of CD24 - already present in undifferentiated cells - Claudin1 and GGT1 was evident throughout the differentiation protocol (Fig. 2c). We next performed gene expression analysis by real-time PCR and found that the expression of gene and corresponding protein paralleled over time (Fig. 2d). Collectively, our results demonstrate that the protocol we established supports the efficient commitment of the two different human iPSC cell lines towards RPCs and provides the basis for functional studies.

**Figure 3:**
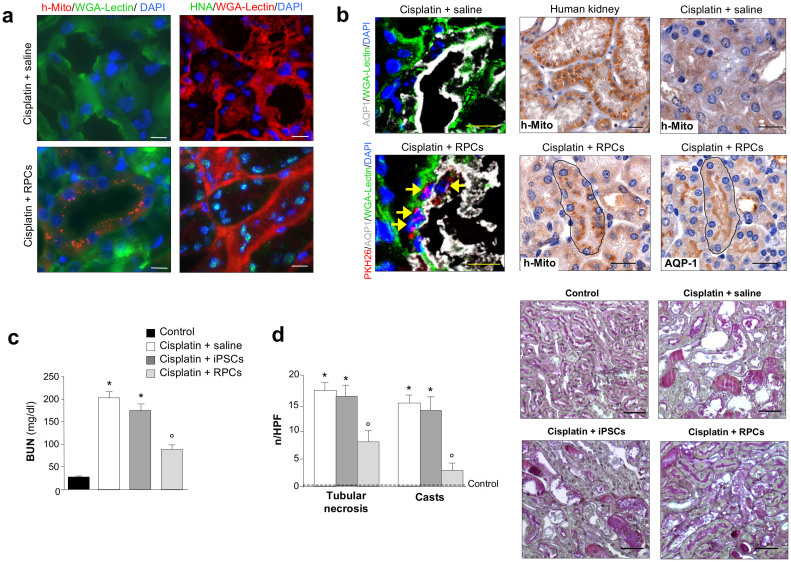
Human iPSC-derived RPC regenerative potential in experimental AKI. (a) Human iPSC-derived RPCs identified in mice with cisplatin-induced AKI by human mitochondrial (h-Mito) staining (red) or HNA staining (green). Scale bars: 10 μm. (b) Human iPSC-derived RPCs, pre-labelled with PKH26 cell tracker (red, arrows) identified in cisplatin mice, express AQP1 (white, left panels). Immunoperoxidase staining of renal sections of cisplatin mice receiving saline or iPSC-derived RPCs (serial sections) labelled with h-Mito or AQP1 antibody (central and right panels). In serial sections, the areas encircled by a black line represent the same tubule. Human kidney section is shown as positive control for h-Mito staining. Scale bars: 20 μm. (c) Renal function expressed as blood urea nitrogen (BUN) in mice with cisplatin induced AKI given saline, undifferentiated iPS cells (iPSCs) or iPSC-derived RPCs at 4 days (mean ± SEM, n = 5, *p < 0.01 versus Control; °p < 0.01 vs cisplatin plus saline or iPSCs). (d) Histological evaluation of kidney samples from control, cisplatin mice receiving saline, undifferentiated iPS cells or iPSC-derived RPCs, 4 days after cisplatin (mean ± SEM, n = 5, *p < 0.01 versus Control; °p < 0.01 vs cisplatin plus saline or iPSCs). Scale bars: 50 μm.

The derivation of RPCs from iPSCs offers new platforms for drug screening and for *in vitro* tissue engineering however, the major challenge resides on their employment in regenerative medicine to *in vivo* generate new functional cells. Therefore, we next assessed the renoprotective potential of human iPSC-derived RPCs, obtained on day 12, in the murine model of cisplatin-induced AKI which is characterized by severely compromised renal function and tubular damage 4 days after drug injection^30^. Intravenously infused iPSC-derived RPCs were found in the kidney as early as 24 h. At 4 days, a sustained number of iPSC-derived RPCs was observed in the cortical region of the kidney of cisplatin mice as proved by the presence of human mitochondria (h-Mito), human nuclear antigen (HNA) or PKH26 cell tracker (Fig. 3a and b). Human cells were found integrated into murine tubuli identified by wheat germ agglutinin (WGA)-lectin staining and cortical areas showed extensive engraftment accounting for 42% ± 12 HNA positive cells per high power field (HPF). By immunofluorescence staining with AQP1, we provided evidence that human PKH26 positive iPSC-derived RPCs mainly engrafted in proximal tubuli and expressed a marker of proximal tubular cells (Fig. 3b, left). This result was confirmed by immunoperoxidase technique showing the co-localization of the signal for human mitochondria and AQP1 in proximal tubular cells (Fig. 3b, central and right panels). No human iPSC-derived RPCs were found in peanut agglutinin (PNA)-lectin positive distal tubuli while scattered human cells were observed in AQP3-positive collecting ducts. Engrafted human iPSC-derived RPCs, co-stained for the proliferation marker Ki-67 and HNA, showed 30% of human cells proliferating in proximal tubuli (Supplementary Fig. 3). Tropism of transplanted human cells for the kidney was supported by the observation that a paltry number of engrafted cells was found in other organs including liver (0.2 ± 0.07 cells/HPF), lung (0.26 ± 0.06 cells/HPF), heart (0.22 ± 0.08 cells/HPF) and spleen (0.16 ± 0.06 cells/HPF) 24 h after cell infusion (Supplementary Fig. 4) while no human cells were found at 4 days in all the organs studied. Mice that experienced renal engraftment exhibited a significant improvement of renal function to the extent of a 55% reduction of blood urea nitrogen (BUN) levels as compared with cisplatin-treated mice given saline (Fig. 3c). Renal histology of mice receiving human iPSC-derived RPCs was significantly improved, as shown by the remarkable reduction of renal tubular damage in terms of cell swelling, cast deposition, loss of brush border and cell necrosis compared to mice receiving saline (Fig. 3d). Proliferation of tubular cells, assessed by Ki-67 expression, was increased primarily in the proximity of engrafted human cells compared to cisplatin mice given saline (Ki-67 positive cells/HPF: iPSC-derived RPCs, 14 ± 0.86 vs saline, 8 ± 0.06; p < 0.01).

Injection of human undifferentiated iPSCs, here used as control, failed to exert any protective effect on renal function (Fig. 3c) and renal histology (Fig. 3d). Undifferentiated iPSCs were not present in the kidney and in the other organs at both 24 h and 4 days after cisplatin injection. No signs of inappropriate differentiation and tumor formation by iPSC-derived RPCs were noted in the renal tissues either at 4 days or 8 weeks after cisplatin injection.

Herein, we defined a successful differentiation protocol to efficiently derive renal progenitor cells from human iPSCs that consisted of the exposure of the cells to specific small molecules and growth factors resulting in the acquisition of markers sequentially expressed in different phases of kidney development. During the first stage of the differentiation process, we obtained a cell population with widespread expression of IM markers, whereas later on cells acquired MM phenotype consistent with the presence of early renal progenitor cells and also expressed markers such as NCAM and CD24 in a specialized cell-cell contact distribution. This achievement represents a new platform to push the research of patient-specific pluripotent stem cells toward the generation of renal tissues. The capacity of human iPSC-derived RPCs to exert renoprective effects has been proved here in a murine model of AKI. The intravenous cell administration is compatible with possible future extension of this approach to human patients. Moreover, we have additionally found that iPSC-derived RPCs extensively engraft damaged tubuli, proliferate and promote proliferation of adjacent resident tubular cells, acquire tubular epithelial phenotype and improve renal function and tubular injury. Whether the reparative properties of human iPSC-derived RPCs are dependent on direct incorporation into damaged renal structure or via the activation of tubular cell proliferation is open to speculation. Although efforts must ensue for successful translation to patients, this knowledge points the way for the treatment of kidney diseases by means of stem cell-based technologies and fulfills the promise of the therapeutic value of iPSCs. The successful generation of patient-specific iPSC-derived renal progenitors will enable diverse biomedical applications including developmental studies, high-throughput drug discovery as well as providing physiologically relevant cells for disease modelling.

## Methods

### Cell culture

Commercially available human iPSCs (SC101A-1) were purchased from System Biosciences International and maintained in DMEM/F12 medium containing 20% Knockout Serum Replacement (Invitrogen), 0.1 mM nonessential amino acids (Invitrogen), 0.1 mM 2-mercaptoethanol (Sigma), 10 ng/ml human basic fibroblast growth factor (FGF2, PeproTech) and 1% penicillin and streptomycin. Mouse embryonic fibroblasts (MEFs), used as feeder cells for iPSCs, were isolated from embryos of 13.5 days (E13.5) from CD1 mice and cultured in DMEM medium containing 10% FBS (Invitrogen), 0.1 mM nonessential amino acids (Invitrogen) and 1% penicillin and streptomycin. For passaging, human iPSCs were incubated with accutase (Millipore). Cell suspension was seeded on mitomycin C-inactivated MEFs in the presence of Rock inhibitor (Sigma). Human dermal neonatal fibroblast cells (NHDF- Neo) were purchased from Lonza and were maintained in MEM medium supplemented with 10% FBS (Lonza), 2 mM Glutamine (Invitrogen), 1 mM Sodium Pyruvate (Invitrogen) and 1% penicillin and streptomycin.

### Derivation and characterization of human iPS clone IV

#### Generation of human iPS cell line

Human iPSCs were derived from human dermal neonatal fibroblasts by STEMCCA Cre-Excisable Constitutive Polycistronic (OKMS) lentivirus (Millipore) following manufacturer's instructions. Briefly, a total of 25,000 human neonatal fibroblasts were plated on a plastic 35-mm culture plate. The next day cells were infected with 200 MOI of virus in human fibroblast medium in the presence of polybrene (5 μg/ml). The medium was replaced 24 hours after transduction with fresh virus-containing medium for a second cycle of infection. Six days after the first infection fibroblasts were trypsinized and plated at different densities on MEF-feeder coated dishes (eg. 20,000 or 50,000 per 10-cm culture dish). The next day the medium was changed to human iPSC-medium. The medium was changed every other day until the first colonies started to appear and then every day. iPSC colonies were manually picked about 30 days post-infection, transferred into 0.5 ml of human iPSC-medium and dissected to small clumps by pipetting and seeded onto a new MEFs feeder in a well of six well-plate. The colonies were mechanically split for 3 passages and then cultured as described above. Clone IV has been chosen for characterization and differentiation studies.

#### Karyotyping

Metaphase spreads were prepared after treatment with 10 μg/ml Colcemid and processed for karyotype analysis. At least 20 metaphases of each sample were counted. Karyotype analysis was performed in collaboration with the Genetic Medicine Laboratory of Azienda Ospedaliera Papa Giovanni XXIII, Bergamo (Italy).

#### Embryoid body (EB) formation

For EB formation, human iPSCs clone IV were harvested by treating with DMEM/F12 containing 1 mg/ml collagenase IV at 37°C. When the perimeter of the colonies appeared highlighted and the cells started to peel up collagenase was removed and the colonies were scraped in human iPSCs medium and centrifuged. The clumps of the cells were suspended and cultured in DMEM/F12 medium supplemented with 20% Knockout serum replacement, 0.1 mM nonessential amino acids, 0.1 mM 2- mercaptoethanol and 1% penicillin and streptomycin and then transferred to low attachment dish. The medium was replaced every other day. After 8 days as floating culture, EBs were transferred onto gelatin-coated plate and cultured in the same medium for another 8 days. At day sixteen the plates were fixed with 4% PFA solution and processed by immunostaining.

#### Teratoma formation and analysis

Undifferentiated human iPS clone IV (10^6^/site) were injected subcutaneously into dorsal flank of NOD-SCID mice (n = 3). Eight weeks after injection, teratomas were dissected, fixed in formalin, embedded in paraffin and stained for hematoxylin and eosin.

### Human iPSC differentiation into renal progenitor cells

To induce differentiation toward mesoderm (ME) and intermediate mesoderm (IM)-like cells human iPSC-growth medium was replaced with basal DMEM/F12 medium containing 5% FBS (Hyclone), 0.1 mM nonessential amino acids, 0.1 mM 2- mercaptoethanol supplemented with 0.1 μM All-Trans Retinoic Acid (Sigma), 1 μM CCG1423 (RhoA Inhibitor, Vinci Biochem) and 5 μM LY294002 (PI3K inhibitor) for 6 days. Ten ng/ml Activin A (Prepotech) was added for two days starting on day 2. To induce metanephric mesenchyme (MM) specification and renal progenitors phenotype by day 6 up to 19 the medium was replaced with fresh basal medium containing nephrogenic factors: 50 ng/ml BMP7 (Prepotech), 10 ng/ml FGF2 and 15 ng/ml GDNF (Abcam). The medium was changed every 2 days.

### RNA Isolation and real-time PCR assays

Total RNA was isolated by RNeasy plus Mini Kit (Qiagen). First-strand cDNA was produced from 2 μg total RNA using SuperScript II First-Strand Synthesis Systems Kit (Invitrogen) using a mixture of oligo-dT and random hexamers oligonucleotides following manufacturer's instructions. The pluripotency of human iPSCs clone IV was evaluated by TaqMan gene expression assay (Life Technologies) using predesigned probes for pluripotent and undifferentiated gene markers (Supplementary Table 1) according to the supplier's instructions. RNA from commercially available human iPSCs was used as reference sample and the gene expression level was normalized to RAF1 housekeeping gene.

For gene expression analysis in renal induction experiments TaqMan Array Human Endogenous Control Plate (Life Technologies) was used in order to evaluate the best housekeeping gene and ELF1 gene was chosen to normalize the gene expression level. Real-time PCR assays were performed using Power Syber Green PCR master mix (Life Technologies). The primers employed are listed in Supplementary Table 2. The ΔΔCt technique was used to calculate cDNA content in each sample using the cDNA expression of the pluripotent state (d0) as a calibrator. The experiments were performed in triplicate and the results were expressed as mean ± SD.

### Cell immunostaining

Cells were fixed in 4% PFA in PBS for 30 min at room temperature. When appropriate, cells were permeabilized in 0.5% Triton X-100 for 30 min at room temperature followed by incubation in blocking solution (5% BSA). The primary antibodies were diluted according to manufacturer's recommendations and incubated overnight at 4°C, followed by incubation with the appropriate secondary antibody for 1 h at room temperature. Primary antibodies included anti-OCT4 (Santa Cruz); anti-NANOG (Santa Cruz); anti-TRA-1-60 (Millipore); anti-TRA-1-81 (Millipore); anti-SSEA3 (Santa Cruz); anti-SSEA4 (Santa Cruz); anti-AFP (Santa Cruz); anti-α-SMA Cy3- conjugated (Sigma); anti-β III Tubulin Alexa Fluor 488- conjugated (Millipore), anti-T (Abcam), anti-Osr1 (Santa Cruz), anti-Lhx1 (Abcam), anti-Wt1 (R&D System), anti-Pax8 (Novus biologicals), anti-Six2 (Proteintech, Abnova), anti-Pax2 (Invitrogen), anti-Sall1 (R&D System), anti-CD24 (Santa Cruz), anti-NCAM (Millipore), anti-AQP1 (Abcam), anti-GGT1 (Santa Cruz), anti-Claudin1 (Thermo Scientific), anti-Pax6 (Novus biologicals) and anti-Nkx2.5 (Thermo Scientific). For CD133 staining, cells, after fixing, were blocked with 1.5% normal goat serum for 1 h at RT and incubated with anti-CD133 antibody (Miltenyi Biotech, AC133/1) for 1 h at 37°C. Subsequently the cells were washed twice with 0.1% Tween 20 and twice with PBS. Appropriate Alexa Fluor 546 and Alexa Fluor 488 secondary antibodies (Molecular Probes) were used followed by nuclear counterstaining with 4′,6-diamidino-2-phenylindole (DAPI) for 10 minutes at room temperature. Images (representative of n = 3 experiments) were taken by Apotome Axio Imager Z2 (Zeiss).

### Flow cytofluorimetric analysis

TRA-1-81 expression was performed by flow cytometer FACs Canto (BD Bioscience). Cells were detached and incubated for 30 min with 1% BSA. Cells were then stained for 1 h with TRA-1-81 (Abcam), primary antibody diluted according to manufacturer's recommendations followed by the appropriate secondary antibody.

### *In vivo* experiments

Animal care and treatment were in accordance with institutional guidelines in compliance with national (D.L. n.116, G.U., suppl 40, 18 February 1992, Circolare No. 8, G.U., 14 July 1994) and international laws and policies (EEC Council Directive 86/609, OJL 358, Dec 1987; NIH Guide for the Care and Use of Laboratory Animals, U.S. National Research Council, 1996). Animal studies were submitted to and approved by the Institutional Animal Care and Use Committee of “Mario Negri” Institute (Milan, Italy). Animals were housed in a constant temperature room with a 12:12-h dark-light cycle and fed a standard diet.

### Cisplatin-induced AKI mouse model and iPSC injection

Two-month-old female NOD-SCID mice (Charles River Italia S.p.a.) received a subcutaneous injection of 13.9 mg/kg cisplatin (Ebewe Italia Srl) and after 24 h were divided into three groups receiving an intravenous injection, through the tail vein, of saline, undifferentiated iPSCs or iPSC-derived RPCs (5 × 10^5^ cells/mouse). In selected experiments, iPSC-derived RPCs have been pre-labeled with cell tracker PKH26 (Sigma Aldrich) following manufacturer's instructions. Mice were sacrificed 4 days after cisplatin and kidneys were used for histology and immunohistochemistry. Human cell engraftment in kidney, liver, lung, heart and spleen was evaluated in cisplatin-treated mice 24 hours after infusion of iPSCs or iPSC-derived RPCs. Renal function was assessed as blood urea nitrogen (BUN) by the Reflotron test (Roche Diagnostics Corporation). BUN levels exceeding 30 mg/dl were considered abnormal. To evaluate any possible cell maldifferentiation or tumor formation, an additional group of cisplatin-mice injected with iPSC-derived RPCs (n = 3 animals) were sacrificed 8 weeks after cisplatin injection and kidney, heart, liver and lung were examined. Normal, untreated mice served as controls.

### Immunohistochemical analysis of tissues from kidney and other organs

Kidney samples were fixed in Duboscq-Brazil and paraffin sections were stained with hematoxylin and eosin, or periodic acid-Schiff's reagent (PAS). Luminal hyaline casts and tubular necrosis (denudation of tubular basement membrane) were assessed in non-overlapping fields (up to 28 for each section) (40X, high power field, HPF).

To identify human iPSCs in murine renal tissues, acetone-fixed cryosections were incubated with anti-human nuclei antigen (HNA) Alexa Fluor 488 (Millipore) or anti human mitochondria (Millipore) primary antibody diluted according to manufacturer's recommendations followed by Cy3-conjugated donkey anti-mouse antibody (Jackson Laboratories). Sections were then co-stained with Rhodamine or FITC-labeled wheat germ agglutinin and DAPI. Fluorescence was examined by Apotome Axio Imager Z2 (Zeiss). The percentage of HNA positive cells per field was calculated by counting HNA- positive cells on total DAPI positive nuclei in 20 non-overlapping random areas per section. Human iPSC-derived RPCs, pre-labeled with PKH26, have been studied in PLP fixed renal sections co-stained with anti-AQP1 antibody (Abcam) followed by the appropriate Cy5-conjugated secondary antibody, FITC-WGA lectin and DAPI staining. In additional experiments, to identify human cells in distal tubuli and collecting ducts, sections have been stained with anti PNA-lectin (Vector Laboratories) or anti-AQP3 antibody (AbCam), respectively.

Duboscq-Brazil-fixed, paraffin-embedded kidney sections (3 μm) were used to study immunophenotype of human cells in the kidney of cisplatin-injected mice. Briefly, sections were deparaffinized, rehydrated, and then incubated for 30 minutes with 0.3% H_2_O_2_ in methanol to quench endogenous peroxidase. After antigen retrieval and blocking with 1% bovine serum albumin (BSA), serial sections were incubated with the primary antibodies rabbit AQP1 (Santa Cruz) or anti human mitochondria (Millipore). Then, specimens were incubated with the biotinylated species-specific secondary antibodies, avidin-biotin peroxidase complex solution, and developed with diaminobenzidine. Slides were finally counterstained with hematoxylin, dehydrated in graded alcohols, mounted and observed by light microscopy (ApoTome Axio Imager Z2, Zeiss). Negative controls were obtained by omitting the primary antibody on adjacent sections. Human kidney tissues have been used as positive controls for human mitochondria staining.

In order to study cell proliferation of human iPSC-derived RPCs in the kidney 4 days after cisplatin treatment, acetone-fixed cryosections were incubated with anti-HNA-Alexa fluor 488 (Millipore) and anti-Ki67 (Abcam), followed by the appropriate Cy5-conjugated secondary antibody, rhodamine-labeled WGA lectin and DAPI. Cell proliferation has been quantified as the number of Ki-67-positive cells/HPF (25–30 fields/section, n = 4 animals/group).

Human iPSCs and iPSC-derived RPC engraftment was analyzed in formalin-fixed, paraffin-embedded liver, lung, heart and spleen. Three sections for each animal (n = 3/group) have been stained with anti-human mitochondria (Abcam) as previously described. RPC engraftment was quantified as the number of human mitochondria-positive cells/HPF (20–35 fields/section).

### Statistical analysis

Results are expressed as mean ± SEM. Data were analyzed by ANOVA followed by Tukey-Cicchetti test for multiple comparisons or *t* test for unpaired data, as appropriate. *P* < 0.05 was considered to represent statistically significant differences.

## Author Contributions

B.I.: conception and study design, iPSCs maintenance and differentiation protocols, immunofluorescence analysis, data analysis and interpretation and writing the manuscript; S.T., O.C. and M.D.: iPSCs Clone IV derivation and characterization, iPSCs maintenance and differentiation protocols, immunofluorescence analysis, real-time PCR, data analysis and interpretation; A.P.: iPSCs maintenance and differentiation protocols, immunofluorescence analysis, in vivo experiments in AKI model; C.X.: data analysis and interpretation; E.P.: iPSCs maintenance and differentiation protocols, immunofluorescence analysis, data analysis and interpretation; R.N., P.R.: immunohistochemical analysis; A.B.: conception and study design, data analysis and interpretation; G.R.: data analysis and interpretation, writing the manuscript; M.M.: conception and study design, data analysis and interpretation and writing the manuscript.

## Supplementary Material

Supplementary Information Supplementary information
