# Supplementary material for: Renal progenitors derived from human iPSCs engraft and restore function in a mouse model of acute kidney injury
Source: Sci Rep. 2015 Mar 6;5:8826. doi: 10.1038/srep08826 (PMC4351529; doi:10.1038/srep08826)
Supplement: Supplementary Information [file srep08826-s1.doc]

**Supplementary Information**

**Renal progenitors derived from human iPSCs engraft and restore function in a mouse model of acute kidney injury**

Barbara Imberti, Susanna Tomasoni, Osele Ciampi, Anna Pezzotta, Manuela Derosas, Christodoulos Xinaris, Paola Rizzo, Evangelia Papadimou, Rubina Novelli,

Ariela Benigni, Giuseppe Remuzzi and Marina Morigi

**CORRESPONDING AUTHOR**

Ariela Benigni, PhD

IRCCS - Istituto di Ricerche Farmacologiche Mario Negri

Centro Anna Maria Astori

Science and Technology Park Kilometro Rosso

Via Stezzano 87, 24126 Bergamo, Italy

Tel: +39 035-42131; Fax: +39 035-319331

Email: ariela.benigni@marionegri.it

**Supplementary Figure 1. Characterization of human iPSC clone IV generated by Cre-excisable polycistronic lentiviral vector.**

**
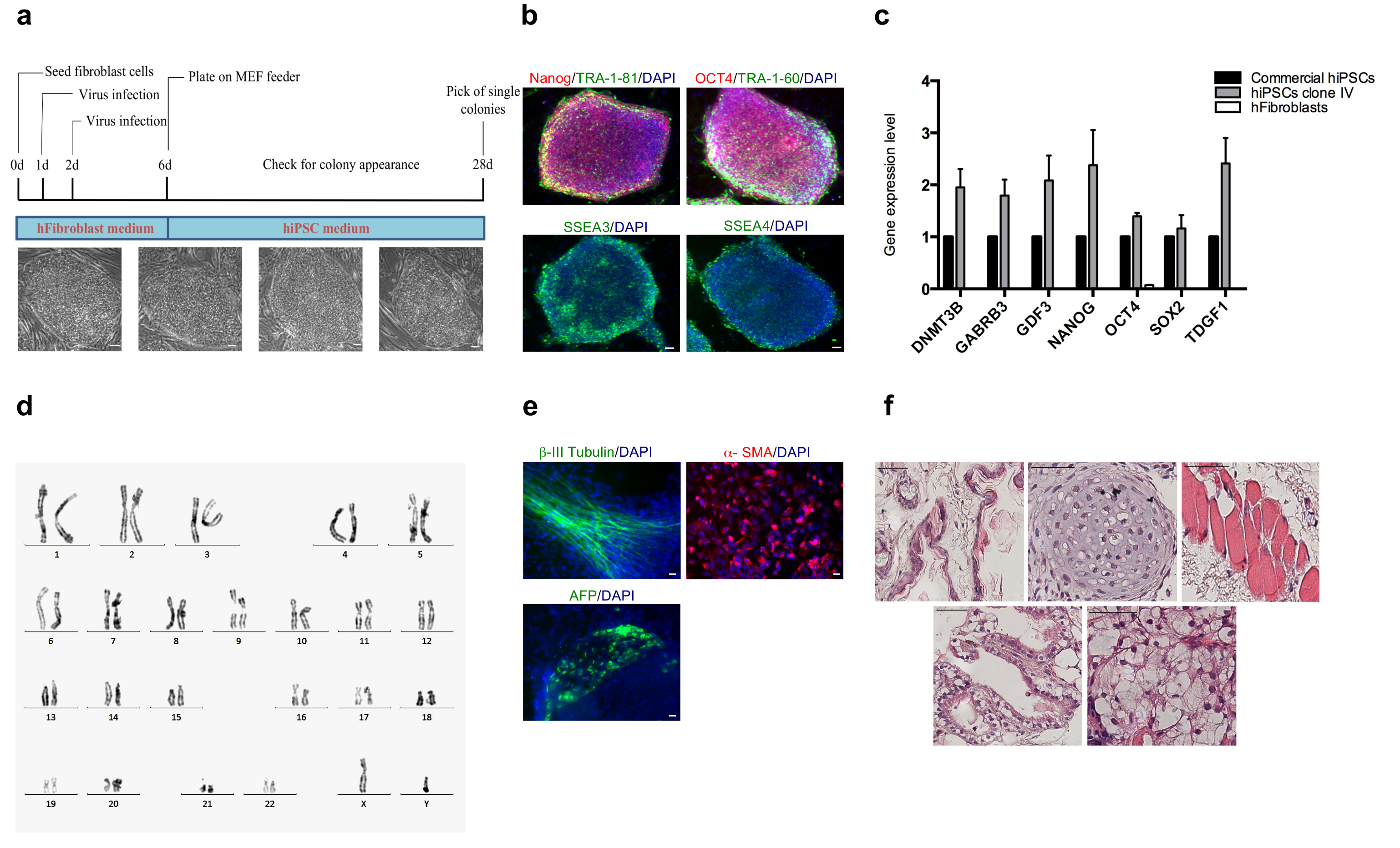
**

**(a**) Time course of reprogramming experiments and stereomicrographs of human iPSC clones obtained. (**b**) Immunofluorescence analysis showing expression of the pluripotency markers. **(c**) Gene expression level of pluripotency markers compared to commercially human iPSCs set as reference sample and to parental fibroblasts. **(d**) Karyotype analysis revealing normal chromosomal number and structure. (**e**) *In vitro* differentiation through embryoid bodies formation: β−III Tubulin (ectoderm), AFP (endoderm), α-SMA (mesoderm). (**f**) Hematoxylin-eosin staining of teratomas: (upper images, from left to right) keratin containing epidermal tissue (ectoderm), cartilage (mesoderm), striated muscle (mesoderm); (lower images, left and right respectively) gut-like epithelium (endoderm), adipose tissue (mesoderm). Scale bars: 50 μm (a, b, e, f).

**Supplementary Figure 2. Renal differentiation of human iPSCs clone IV.**


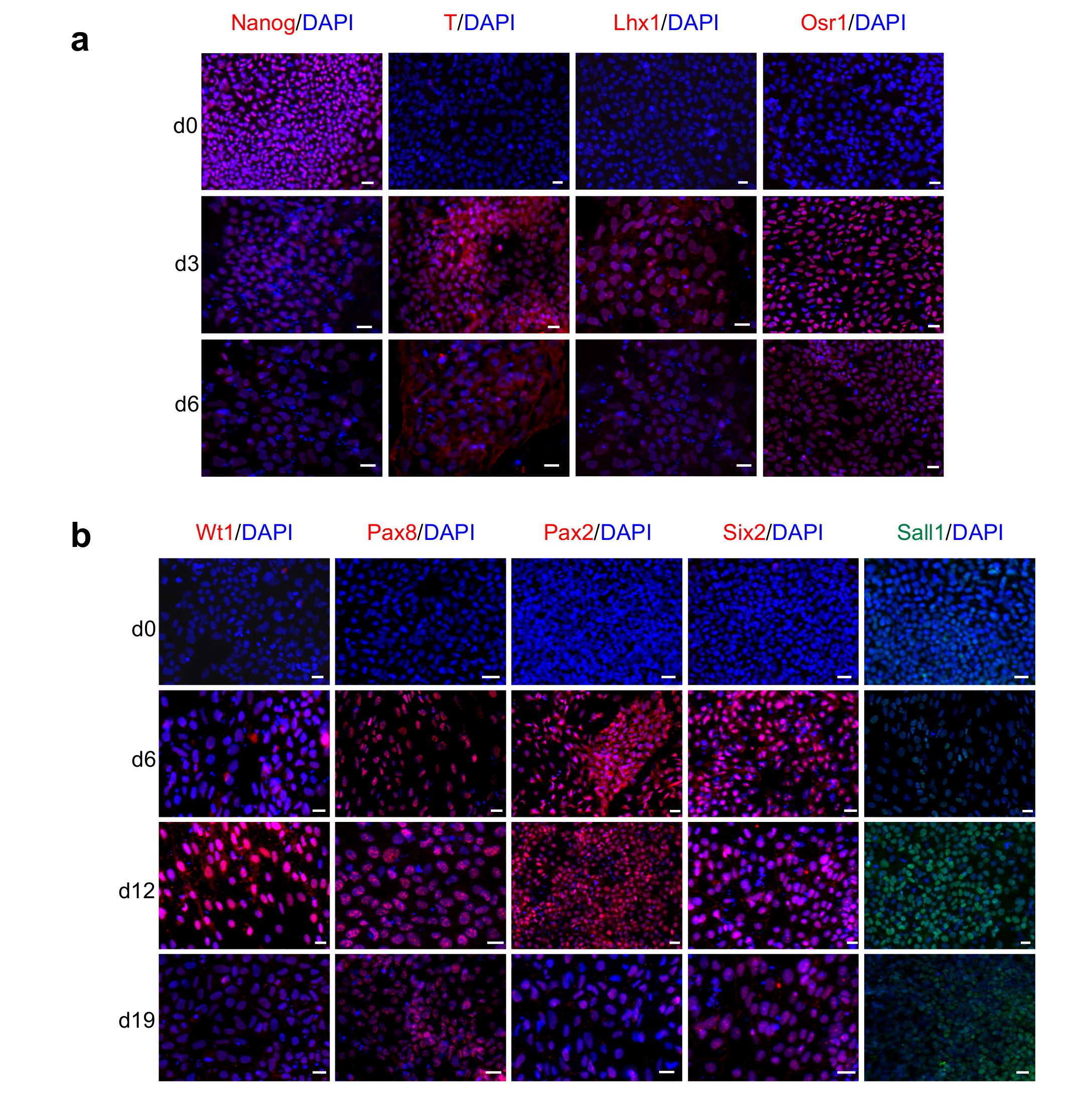


**(a**) Representative images of immunofluorescence staining for Nanog, T, Lhx1 and Osr1 in human iPSCs clone IV exposed to differentiation protocol up to day 6. (**b**) Immunofluorescence analysis of Wt1, Pax8, Pax2, Six2 and Sall1 in cells exposed to inductive medium at different time points. Nuclei are stained with DAPI (blue). Scale bars: 20 μm (a, b).

**Supplementary Fig. 3. Cell proliferation in renal tissue of mice with cisplatin-induced AKI**


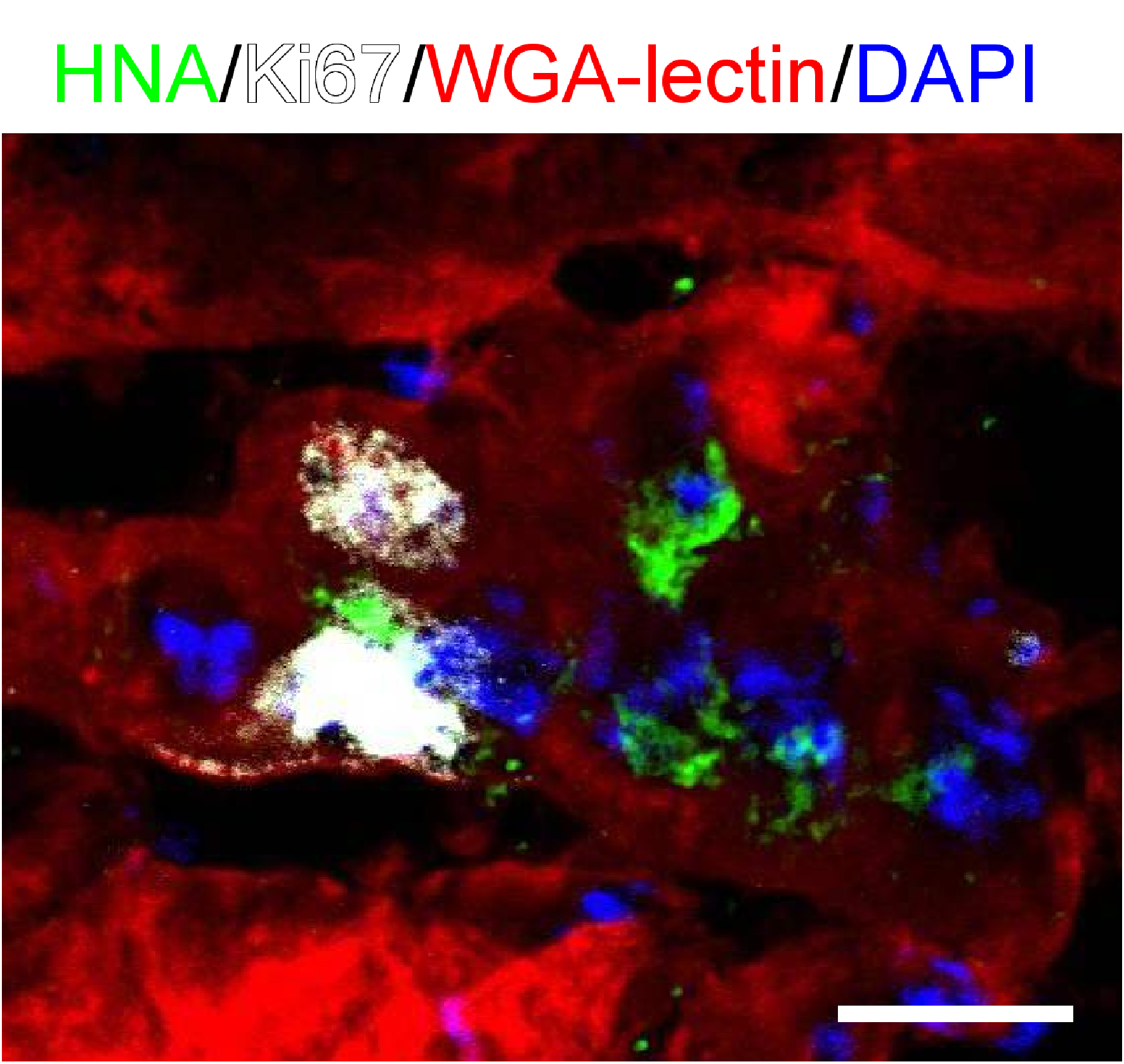


Representative immunofluorescence image of renal tissue of a mouse with AKI at 4 days showing Ki-67 positive proliferating cells (white). Human iPSC-derived RPCs are labelled for human nuclear antigen (HNA, green). Renal structure is labelled with wheat germ agglutinin (WGA, red) and nuclei are stained with DAPI (blue). Scale bars: 10 μm.

**Supplementary Fig. 4. Human iPSC-derived RPC distribution in different organs 24 hours after cell infusion in experimental AKI mice**


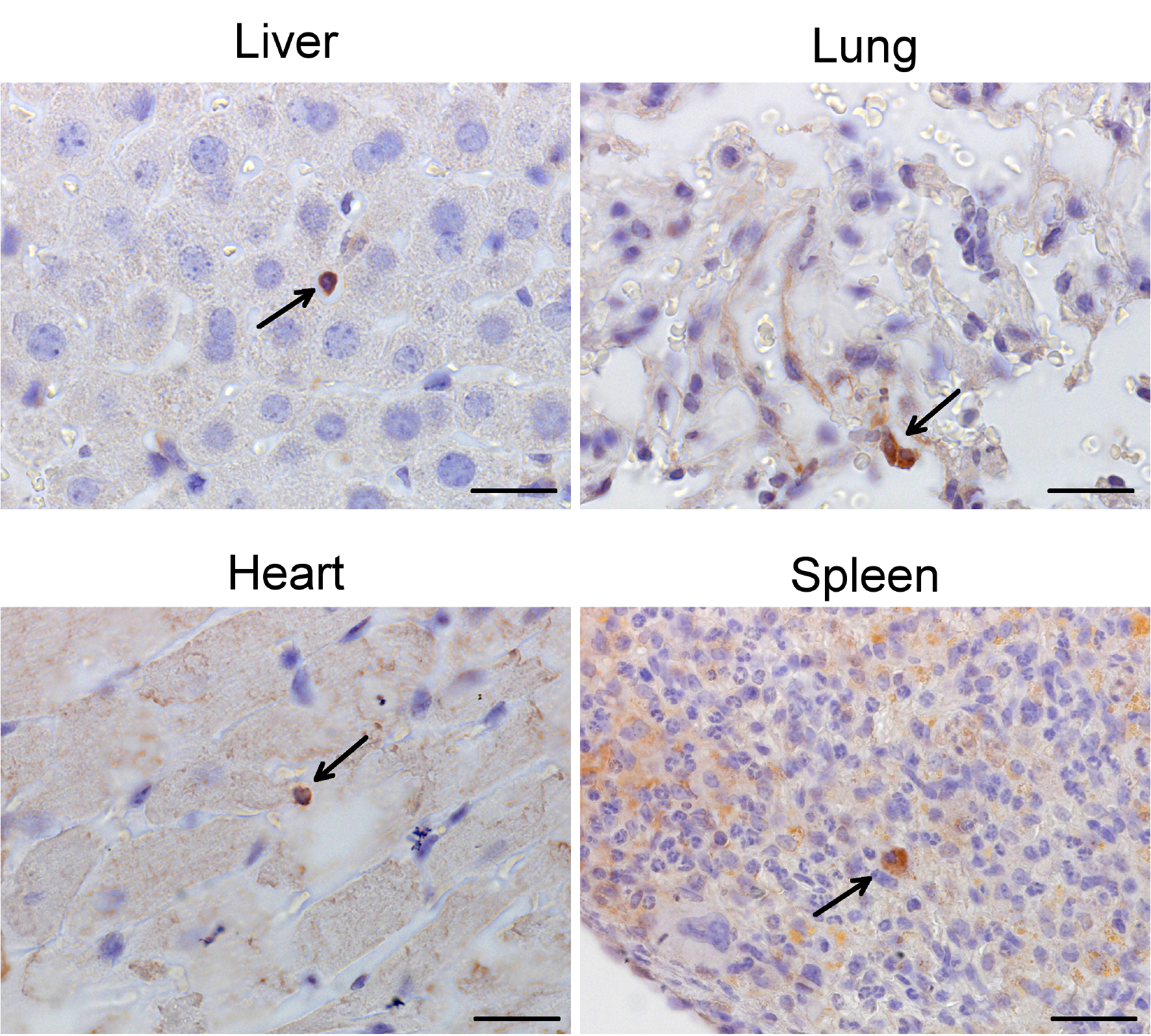


Representative immunoperoxidase images of iPSC-derived RPCs labelled for human mitochondria (arrows) in liver, lung, heart and spleen of a mouse with cisplatin-induced AKI at 24 hours after cell injection. Scale bars: 20 μm.

**Supplementary Table 1. List of TaqMan probes**

| **Catalog number** | **Gene Symbol** | **Description** |
| --- | --- | --- |
| Hs00742896_s1 | POU5F1 | Homo sapiens POU class 5 homeobox 1 (OCT4) |
| Hs02387400_g1 | Nanog | Homo sapiens Nanog homeobox |
| Hs00171876_m1 | DNMT3B | Homo sapiens DNA (cytosine-5-)-methyltransferase 3 beta |
| Hs00241459_m1 | GABRB3 | Homo sapiens gamma-aminobutyric acid (GABA) A receptor, beta 3 |
| Hs00220998_m1 | GDF3 | Homo sapiens growth differentiation factor 3 |
| Hs00602736_s1 | SOX2 | Homo sapiens SRY (sex determining region Y)-box 2 |
| Hs02339499_g1 | TDGF1 | Homo sapiens teratocarcinoma-derived growth factor 1 |
| Hs00234119_m1 | RAF1 | v-raf-1 murine leukemia viral oncogene homolog 1 |

**Supplementary Table 2. Primer Sequences**

| **Gene** | **Accession number** | **Forward sequence** | **Reverse sequence** |
| --- | --- | --- | --- |
| hELF1 | NM_172373.3 | CTTCCCCAATCTACAGGAGCC | TCTCTCAAGCTTCTTGGCCTTC |
| hT | NM_031944 | CAGTGGCAGTCTCAGGTTAAGAAGGA | CGCTACTGCAGGTGTGAGCAA |
| hLHX1 | NM_005568.3 | CGACTTCTTCCGGTGTTTCG | TGCAGGTGAAGCAGTTCAGG |
| hOSR1 | NM_145260 | CGGAGAGTGAGTGGAGAG | TGAAGCAGATACAGGGATTACA |
| hSIX2 | NM_016932 | CTTGCCACCGTTCATTCT | GGACCAGGACACAGAGTA |
| hPAX8 | NM_013992.3 | GGCTCCACCTCATCCATCAA | CTGCTGCTGCTCTGTGAGTC |
| hNANOG | NM_024865.2 | CAAAGGCAAACAACCCACTT | TCTGCTGGAGGCTGAGGTAT |
| hSALL1 | NM_002968.2 | GTGGACTGCAGCGACCTTTC | TTGTTAGCAACCGGGGCCT |
| hWT1 | NM_001198552.1 | CAGCACAGTGTGTGAACTGC | AGCACCGGTATCTTGTCTTGG |
| hPAX2 | NM_000278.3 | CCCGCAACCCTTCACATCA | CTGAGGGTCCCGCCCA |
